# Carotid atherosclerotic plaque segmentation in multi-weighted MRI using a two-stage neural network: advantages of training with high-resolution imaging and histology

**DOI:** 10.3389/fcvm.2023.1127653

**Published:** 2023-05-24

**Authors:** Ran Li, Jie Zheng, Mohamed A. Zayed, Jeffrey E. Saffitz, Pamela K. Woodard, Abhinav K. Jha

**Affiliations:** ^1^ Mallinckrodt Institute of Radiology, Washington University in St. Louis, St. Louis, MO, United States; ^2^ Department of Biomedical Engineering, Washington University in St. Louis, St. Louis, MO, United States; ^3^ Department of Surgery, Washington University School of Medicine in St. Louis, St. Louis, MO, United States; ^4^ Department of Pathology, Beth Israel Deaconess Medical Center, Boston, MA, United States

**Keywords:** atherosclerotic plaque, MR images, segmentation, CNN, BNN

## Abstract

**Introduction:**

A reliable and automated method to segment and classify carotid artery atherosclerotic plaque components is needed to efficiently analyze multi-weighted magnetic resonance (MR) images to allow their integration into patient risk assessment for ischemic stroke. Certain plaque components such as lipid-rich necrotic core (LRNC) with hemorrhage suggest a greater likelihood of plaque rupture and stroke event. Assessment for presence and extent of LRNC could assist in directing treatment with impact upon patient outcomes.

**Methods:**

To address the need to accurately determine the presence and extent of plaque components on carotid plaque MRI, we proposed a two-staged deep-learning-based approach that consists of a convolutional neural network (CNN), followed by a Bayesian neural network (BNN). The rationale for the two-stage network approach is to account for the class imbalance of vessel wall and background by providing an attention mask to the BNN. A unique feature of the network training was to use ground truth defined by both high-resolution *ex vivo* MRI data and histopathology. More specifically, standard resolution 1.5 T in vivo MR image sets with corresponding high resolution 3.0 T *ex vivo* MR image sets and histopathology image sets were used to define ground-truth segmentations. Of these, data from 7 patients was used for training and from the remaining two was used for testing the proposed method. Next, to evaluate the generalizability of the method, we tested the method with an additional standard resolution 3.0 T in vivo data set of 23 patients obtained from a different scanner.

**Results:**

Our results show that the proposed method yielded accurate segmentation of carotid atherosclerotic plaque and outperforms not only manual segmentation by trained readers, who did not have access to the ex vivo or histopathology data, but also three state-of-the-art deep-learning-based segmentation methods. Further, the proposed approach outperformed a strategy where the ground truth was generated without access to the high resolution ex vivo MRI and histopathology. The accurate performance of this method was also observed in the additional 23-patient dataset from a different scanner.

**Conclusion:**

In conclusion, the proposed method provides a mechanism to perform accurate segmentation of the carotid atherosclerotic plaque in multi-weighted MRI. Further, our study shows the advantages of using high-resolution imaging and histology to define ground truth for training deep-learning-based segmentation methods.

## 1. Introduction

Atherosclerosis is the most common cause of death in the United States and throughout the world (1). Identification of atherosclerotic plaque composition including high risk features such as lipid rich necrotic core (LRNC) with hemorrhage has the potential to allow for event risk assessment and may allow better selection of patients for intervention (2, 3). High-resolution multi-weighted magnetic resonance imaging (MRI) has emerged as an effective tool for visualization and characterization of atherosclerotic plaque composition (4, 5). The signal characteristics of major plaque components across MR sequences of various (T1, T2, proton density) weighting have been well established with respect to histology (6, 7). Five different atherosclerotic plaque components have been identified based on signal intensities of multi-weighted MR images (Table 1). However, the manual segmentation and classification of plaque components, which currently depends on offline processing, requires a time consuming comparison of plaque signal characteristics across at least four sets of differently contrast-weighted MR images. This is labor-intensive, and therefore costly, and has the potential to delay the delivery of medical care. To address these issues, several automated segmentation algorithms based on multi-weighted MR images have been developed (8–14). These are typically supervised segmentation methods that use a data subset as a training set on which segmentation is performed manually. While these methods perform voxel-wise segmentation using image properties such as absolute value of intensities, intensity gradients, and wall distances, most are highly dependent on manually provided reference values. These values are error-prone due to two reasons: (1) inter and intra-reader variability; and (2) training set image quality which, because of its *in vivo* acquisition, often has limited resolution, and suffers from motion and noise-related artifacts. To accurately segment various plaque components, histopathology is the preferred gold standard as the ground truth. However, direct comparison of histopathology and *in vivo* MR images with relatively low spatial resolution is intractable due to the differences and inconsistences in image characteristics, including shrinking size of fixed tissue, different orientation and slice thickness.

**Table 1:** Criteria of tissue segmentation. The symbols describe the signal intensity relative to adjacent muscle.

	TOF	T1W	PDW	T2W
LRNC with				
Older hemorrhage	+	+	+	+
Fresh hemorrhage	+	+	−/O	−/O
No hemorrhage	O	O/+	O/+	−/O
Calcification	−	−	−	−
Fibrous tissue	−	O	O	O

+: hyperintense, O: isointense, −: hypointense.

In this study, we developed a two-stage neural-network-based method for carotid vessel wall and plaque component segmentation by utilizing both high resolution *ex vivo* MR images and histopathology in the same set of patients as ground truth. Use of high-resolution *ex vivo* MR images helps bridge the gap between standard resolution *in vivo* MR images and histopathology images and potentially achieve a more accurate definition of the ground truth. A 9-patient standard resolution 1.5 T *in vivo* MRI data set with corresponding high resolution 3.0 T *ex vivo* MRI data and histopathology images was used to define ground-truth segmentations on the standard-resolution images. Data from 7 of these patients was used to train the proposed two-stage network, while the data from the rest of the 2 patients was used for testing. The first part of the two-stage network was a convolutional neural network (CNN) for inner and outer vessel wall segmentation. The second part was a Bayesian deep neural network (BNN) that allowed for input of aggregated multi-weighted MR image data. The goal of the BNN was to achieve pixel-level segmentation of plaque components. We hypothesize that this two-stage neural network can be used to account for the class imbalance of vessel wall and background and has the potential to out-perform both manual segmentation and state-of-the-art single-stage-based segmentation methods. To evaluate the generalizability of the method to an external dataset, we tested the method on a separate data set of 23 patients 3.0 T *in vivo*-only MR images.

## 2. Method

### 2.1. Data acquisition

A total of 9 patients (6 males and 3 females) who were scheduled for carotid endarterectomy surgery were scanned *in vivo* on a 1.5 T Sonata MR Scanner (Siemens Medical Solutions, Malvern, PA) using bilateral dedicated 4-element carotid surface coils within one week prior to surgery (Machnet, Netherlands). MR sequences acquired spin-lattice relaxation time (T1) weighted, spin-spin transverse relaxation (T2) weighted, proton-density weighted, and time of flight (TOF) images. At approximately 2 h after surgery, the dissected carotid plaque tissue was placed in Phosphate Buffered Saline (PBS) solution and then scanned *ex vivo* on a 3 T Siemens Allegra MR scanner using a similar but higher resolution multi-weighted MR protocol (Table 2a). Note that we also included TOF images in the *ex vivo* acquisition to keep the consistency of contrast (gradient-echo contrast) as those used *in vivo.* A 3.5-cm diameter volume coil (Nova Medical, Inc, Wilmington, MA) was used as a transmitter and receiver (15). After the *ex vivo* MRI examination, the tissue was fixed and stained with hematoxylin and eosin (H&E) and Masson’s trichrome stains. The paraffin-embedded tissue blocks were cut every 1 mm, in an orientation to approximate the orientation of the *vivo and ex vivo* MRI slices. The whole dataset included a total of 84 sets of *in vivo* MR images, *ex vivo* MR images, and corresponding pathological sections. We used the *ex vivo* and pathological sections to establish ground truth.

**Table 2:** MR imaging parameters.

(a) MR imaging parameters used in training and test data sets (9 patients)								
	*In vivo* MR imaging (1.5 T)				*Ex vivo* MR imaging (3.0 T)			
	T1-w	T2-w	PD-w	TOF	T1-w	T2-w	PD-w	TOF
TR/TE(ms/ms)	600/5.6	2,130/56	2,130/5.6	10/2.9	500/10	2,500/40	2,500/10	15/4.9
FOV (mm^2^)	120 × 120	120 × 120	120 × 120	120 × 120	25 × 25	25 × 25	25 × 25	25 × 25
Matrix size	256 × 256	256 × 256	256 × 256	256 × 256	256 × 256	256 × 256	256 × 256	256 × 256
Average number	4	2	2	2	3	2	2	4
Slice thickness (mm)	3	3	3	3	1	1	1	1
Slice number	12	12	12	12	24	24	24	24

(b) MR imaging parameters used in additional *in vivo* test data set (23 patients**)**				
	*In vivo* MR imaging (3.0 T)			
	T1-w	T2-w	PD-w	TOF
TR/TE(ms/ms)	900/8.7	2,500/56	2,130/11	26/4.7
FOV (mm^2^)	140 × 140	140 × 140	140 × 140	140 × 140
Matrix size	256 × 256	256 × 256	256 × 256	256 × 256
Average number	4	4	4	2
Slice thickness (mm)	3	3	3	3
Slice number	14	14	14	14

As per best practices to evaluate deep-learning-based methods (16) and to evaluate the generalizability of our method to variation in scanners, we also evaluated our method on an additional external dataset acquired with 3 T PET-MRI system. More specifically, an *in vivo* multi-contrast MR images from 23 patients (12 males and 11 females) scanned on a 3.0 T Siemens PET-MRI system (Siemens mMR, Siemens Healthineers, Malvern, PA) were obtained (Table 2b). A pair of 4-element surface coils (Siemens Healthineers, Malvern, PA) was placed around the neck area for signal reception. A total of 445 *in vivo* MR slices with distinguishable carotid anatomy were selected for further analysis.

### 2.2. Data preprocessing and approach to define ground truth

The acquired images were corrected for coil sensitivity using contrast-limited adaptive histogram equalization algorithm (17). In addition, to alleviate the issue of low signal-to-noise ratio, a block-match and 3D filtering algorithm (18) was used to decrease noise prior to segmentation. Images from different MR sequences were co-registered based on the distance to the bifurcation. Following these steps, to generate ground-truth data from data sets of 9 patients for training the network, three atherosclerotic plaque components, namely LRNC with older hemorrhage (late subacute or late chronic hemorrhage >1 week), calcification, and fibrous tissue were segmented. A reader with over five years’ experience in MR imaging was employed to generate ground truth. Intensity-based criteria (Table 1) was used for tissue classification (7) to perform preliminary segmentation of these plaque components. First, the lumen and outer boundary of vessel wall were manually identified. To minimize the impact of noise and improve the consistency of manual segmentation, the adjacent sternocleidomastoid muscle was used as a reference to quantitatively define the threshold and signal intensity criterion. The preliminary segmentation was then manually validated with assistance of *ex vivo* images and histopathology to establish the ground truth.

**Figure 1:**
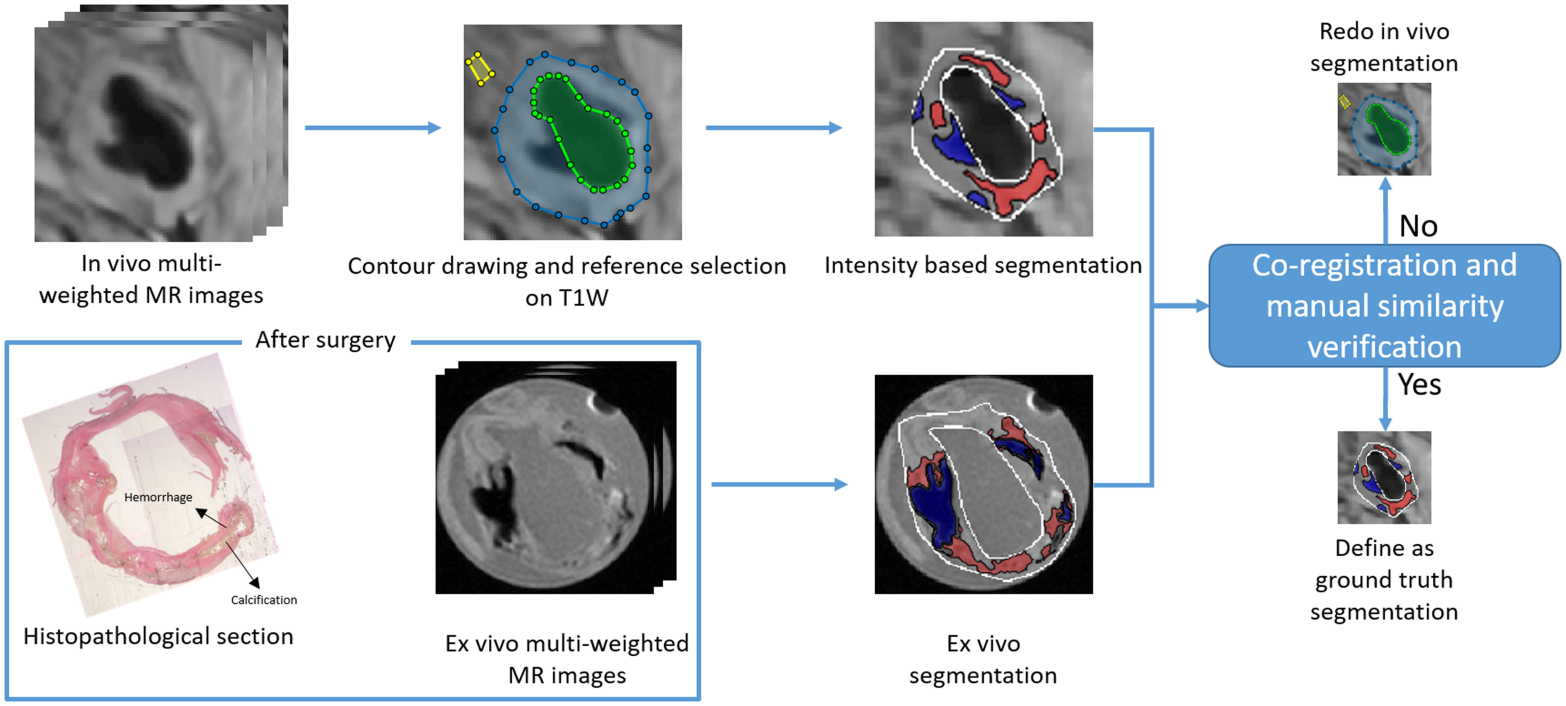
The strategy to generate ground truth segmentation shown on a representative slice.

The segmentation procedure used to generate ground truth is delineated in Figure 1. With slice thickness of only 1 mm in the *ex vivo* images, we could directly compare the segmented *ex vivo* images to the histopathology for a clearer definition of the generated ground truth. We used the same intensity-based criteria as used on the *in vivo* MR images to segment the *ex vivo* image. The second row of Figure 1 shows the histopathological sections with segmentation of their corresponding *ex vivo* MR images. To validate the *in vivo* segmentation, the trained reader ensured that the locations, sizes, and shapes of plaque components in segmented *in vivo* images were as close as possible to the segmented *ex vivo* images. If the *in vivo* segmentation had an apparent difference from *ex vivo* segmentation and pathological sections, the reference muscle was reselected until the difference was eliminated. All ground-truth generating steps were performed using a custom designed tool developed with Matlab. In a total of 84 2D slices acquired from 9 patients, a subset of 70 slices from 7 patients was augmented using flipping and rotation of those images to yield a dataset of 420 slices. This was used as the training set. Once trained, this method was tested with 14 slices from the other 2 patients, where again, the ground truth was defined with the assistance of *ex vivo* MR imaging data and histopathology.

Further, as mentioned above, the method was also tested with an additional test dataset of 445 MR slices from 23 patients obtained from a 3.0 T MR scanner. Ground truth segmentation of the additional test dataset was obtained using manual annotation performed by an experienced observer with the same custom designed tool as described above.

### 2.3. Proposed segmentation method

The proposed method consists of two networks, namely a CNN followed by a BNN, referred to as Stage I and Stage II, respectively. T1W images were used in the CNN algorithm which segmented the contours of lumen and outer artery wall. The output of the CNN was grouped with the 4-channel aggregated MR images and input to the BNN, which then provided segmentation of plaque components. The details of two networks are provided in Supplementary Appendix.

#### 2.3.1. Training

The CNN and BNN were trained separately. We randomly selected 80% of the data out of the whole dataset as the training set. The CNN was trained with T1-weighted images only, and the network hyperparameters were optimized on the training set *via* five-fold cross validation. Subsequently, the BNN was trained with the same training set but comprised of all multi-weighted MR images and vessel wall masks. The hyperparameter combination of BNN was optimized with the same method as that of CNN. The loss functions of the CNN and BNN were a combination of cross-entropy loss, Dice loss and K-L divergence loss, denoted by Loss_CE_, Loss_Dice_ and Loss_KLD_ respectively. These loss functions are given by:

$$\begin{array}{rl} & \mathrm{Los}s_{\mathrm{CE}}=y_{\mathrm{true}}\;\log(y_{\mathrm{pred}})+(1\text{–}y_{\mathrm{true}})\log(1\text{–}y_{\mathrm{pred}})\\ & \mathrm{Los}s_{\mathrm{Dice}}=1\text{–}\frac{2\sum_{\mathrm{pixel}}y_{\mathrm{true}}y_{\mathrm{pred}}}{\sum_{\mathrm{pxiel}}y_{{}_{\mathrm{true}}}^{2}+\sum_{\mathrm{pxiel}}y_{{}_{\mathrm{pred}}}^{2}}\;\\ & \mathrm{Los}s_{\mathrm{KLD}}=\sum_{\mathrm{pxiel}}y_{\mathrm{true}}\log\left(\frac{y_{\mathrm{true}}}{y_{\mathrm{pred}}}\right) \end{array}$$

The mixed loss function of CNN, denoted by Loss_CNN_, is given by:

$$\mathrm{Los}s_{\mathrm{CNN}}=\frac{1}{2}(\mathrm{Los}s_{\mathrm{CE}}+\mathrm{Los}s_{\mathrm{Dice}}).$$

The purpose of the mixed loss function of CNN was to handle the class imbalance caused by the vessel wall, which often occupies a considerably smaller volume relative to the background (19).

The mixed loss function of BNN, denoted by Loss_BNN_, is given by:

$$\mathrm{Los}s_{\mathrm{BNN}}=\frac{1}{2}(\mathrm{Los}s_{\mathrm{CE}}+\mathrm{Los}s_{\mathrm{KLD}}).$$

The Loss_KLD_ was added to the Loss_CE_ for the posterior distribution approximation of the BNN (20).

### 2.4. Data analysis

#### 2.4.1. Figures of merit (FOM) for evaluation

In all the experiments, to evaluate the performance, three FOMs were employed: Dice similarity coefficient (DSC), precision, and sensitivity, given by

$$\begin{array}{rl} & \mathrm{DSC}=\frac{2TP}{FN+FP+2TP}\\ & \mathrm{Sensitivity}=\frac{TP}{FN+TP}\\ & \mathrm{Precision}=\frac{TP}{TP+FP} \end{array}$$

where *TP, TN, FP*, and *FN* denotes true positive, true negative, false positive and false negative of prediction of labels based on normalized signal intensities on multi-weighted MR images respectively. The mean values and 95% confidence intervals (CIs) of these FOMs were computed.

#### 2.4.2. Comparison with other deep learning methods

We compared the performance of the proposed method with three state-of-the-art deep learning methods, namely a U-Net (21), ResNet-101 (22), and DeepLabv3 (23). These methods were chosen as they are widely used in medical image segmentation. All compared methods were trained with ground truth obtained with the assistance of the high-resolution images and optimized *via* five-fold cross validation.

We also compared the performance of our method to performance of trained readers. Two trained readers, each with two years of experience in MR imaging, referred to as Observer I and Observer II, were asked to segment the 14-slice test data using the same standard customized software as we mentioned. These observers were not provided the *ex vivo* data or the histopathologic sections for these 14 slices. Each observer was asked to segment the regions of interest twice to decrease the intra-observer variability. We then compared the performance of each observer with the performance of our proposed method.

In all the comparison studies, statistical significance was assessed *via* a paired sample *t*-test, with a *p*-value <0.05 leading to the inference of a statistically significant difference.

#### 2.4.3. Impact of using histology and *ex vivo* images to define ground truth

To investigate the impact of our high-resolution assisted ground-truth generation procedure on segmentation performance, we trained the proposed method using a strategy where the ground truth was generated without referring to the high-resolution images. In this strategy, one observer was asked to manually annotate all 70 slices of the training set three times to eliminate intra-observer variability. The two-stage neural network was then trained with 3 sets of manually annotated ground truth separately. The performance using this strategy was then evaluated on the test set (14 slices) and compared with the strategy that used high-resolution *ex vivo* MR images and histopathology to define the ground truth.

#### 2.4.4. Sensitivity to variations in training data

We assessed the sensitivity of the plaque segmentation to variations in training data. First, we randomly separated the BNN training data into two subsets. The BNN was trained and optimized on these two subsets individually. This process yielded two versions of the proposed method, each trained with a different dataset. We then evaluated both versions using the 14-slice test data set, resulting in two sets of segmentation. The similarity of these two segmentation sets was quantified using DSC values, with a high value indicating less sensitivity to variations in the training data. For comparison, we also evaluated the sensitivity of the standard U-Net method to this variation in the training data.

#### 2.4.5. Studying the efficacy of using two networks in the proposed method

To study the impact of using two networks in our approach, we compared our method with an approach that just used the BNN (i.e., did not contain the Stage I). Our proposed two-stage method vs. the BNN were trained on the same training set separately and optimized *via* five-fold cross validation.

#### 2.4.6. Evaluation with dataset from different scanner

To evaluate the generalizability of our method to variations in scanners, the proposed method was tested using a test set consisting of 445 3.0 T MR slices described above. An experienced reader with over four years of experience in MR imaging manually annotated 445 multi-weighted MR images using a customized software. Next the trained reader reviewed each preliminary label of the tissue-types and manually corrected inaccurate labels. We also compared the performance of the proposed method on the additional test set with the Standard U-Net, DeepLab v3, and ResNet-101.

## 3. Results

### 3.1. Performance in segmenting plaque components

**Figure 2:**
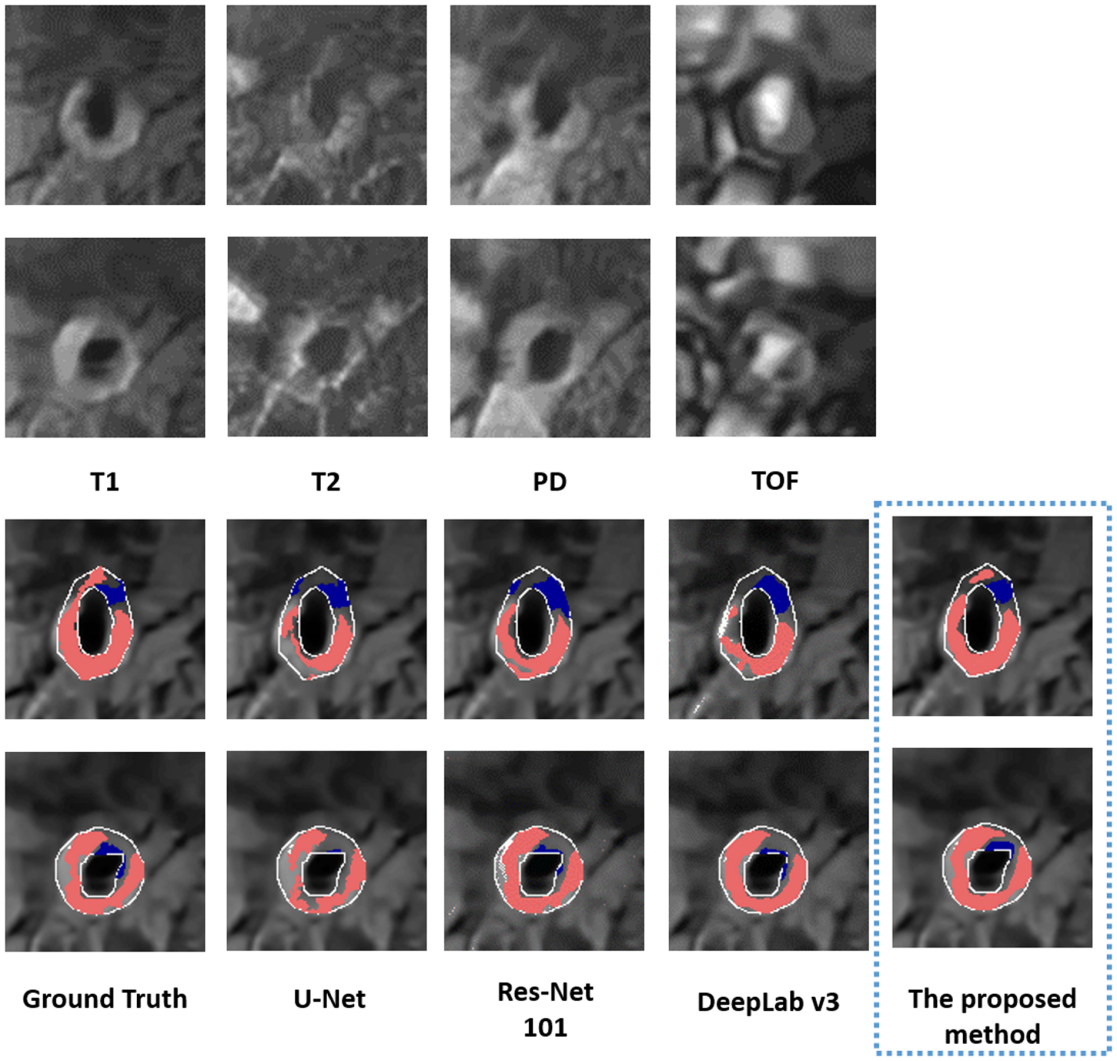
Two representative examples of multi-weighted MR images (acquired at 1.5 T) in the top two rows. The bottom two rows show the segmented LRNC with older hemorrhage (shown in red) and calcification (shown in blue) using the proposed method and three other deep learning-based segmentation algorithms, as compared to the ground truth. We observe that visually, the segmentation predicted by the proposed method is close to the ground truth.

The performance of the proposed method in segmenting each tissue type is shown in Table 3. The proposed method outperformed (*p *<* *0*.*05) all other methods on DSCs of all tissue types, yielding DSCs of 0.78 [95% confidence interval (CI): 0.75, 0.8], 0.62 (95% CI: 0.6, 0.65), and 0.74 (95% CI: 0.73, 0.76) for LRNC with older hemorrhage, calcification, and fibrous tissue, respectively. Two representative results of plaque segmentation are shown in Figure 2.

**Table 3:** Performance in segmenting plaque components (95% confidence interval) in 2/9 patients scanned at 1.5 T MRI.

(a) DSC			
	LR/NC with OH	Calcification	Fibrous tissue
Observer I	0.71 (0.69, 0.72)	0.52 (0.45, 0.58)	0.58 (0.55, 0.6)
Observer II	0.61 (0.59, 0.63)	0.53 (0.52, 0.53)	0.56 (0.5, 0.62)
Standard U-Net	0.7 (0.68, 0.73)	0.49 (0.46, 0.53)	0.67 (0.6, 0.73)
DeepLab V3	0.71 (0.69, 0.73)	0.58 (0.55, 0.61)	0.69 (0.67, 0.71)
ResNet-101	0.75 (0.73, 0.77)	0.56 (0.53, 0.6)	0.65 (0.61, 0.67)
The proposed 2-stage CNN without high-resolution reference	0.76 (0.73, 0.78)	0.56 (0.55, 0.58)	0.64 (0.61, 0.69)
The proposed method	0.78 (0.75, 0.8)	0.62 (0.6, 0.65)	0.74 (0.73, 0.76)
(b) Precision			
	LR/NC with OH	Calcification	Fibrous tissue
Observer I	0.8 (0.79, 0.8)	0.44 (0.43, 0.45)	0.56 (0.51, 0.6)
Observer II	0.77 (0.75, 0.78)	0.38 (0.39, 0.37)	0.54 (0.46, 0.61)
Standard U-Net	0.78 (0.76, 0.8)	0.7 (0.67, 0.74)	0.58 (0.51, 0.65)
DeepLab V3	0.8 (0.78, 0.82)	0.63 (0.6, 0.66)	0.71 (0.69, 0.73)
ResNet-101	0.83 (0.81, 0.85)	0.64 (0.61, 0.68)	0.63 (0.59, 0.65)
The proposed 2-stage CNN without high-resolution reference	0.68 (0.65, 0.7)	0.76 (0.75, 0.78)	0.75 (0.7, 0.82)
The proposed method	0.76 (0.71, 0.8)	0.55 (0.53, 0.58)	0.72 (0.71, 0.74)
(c) Sensitivity			
	LR/NC with OH	Calcification	Fibrous tissue
Observer I	0.64 (0.61, 0.66)	0.68 (0.5, 0.8)	0.58 (0.56, 0.6)
Observer II	0.51 (0.55, 0.64)	0.83 (0.8, 0.85)	0.58 (0.53, 0.62)
Standard U-Net	0.64 (0.62, 0.66)	0.38 (0.35, 0.41)	0.79 (0.75, 0.83)
DeepLab V3	0.64 (0.62, 0.66)	0.54 (0.51, 0.57)	0.67 (0.65, 0.69)
ResNet-101	0.68 (0.66, 0.7)	0.5 (0.47, 0.54)	0.67 (0.63, 0.69)
The proposed 2-stage CNN without high-resolution reference	0.86 (0.83, 0.88)	0.44 (0.43, 0.46)	0.56 (0.53, 0.61)
The proposed method	0.8 (0.79, 0.81)	0.71 (0.69,0.74)	0.76 (0.7, 0.82)

It was observed that the proposed method generally outperformed Observer I and Observer II over a range of tissue types and FOMs (Table 3), yielding 10%–28% higher DSCs in segmenting LRNC with older hemorrhage, calcification, and fibrous tissue, except that Observer II yielded better sensitivity for segmenting calcification. This demonstrates the higher accuracy of our model in comparison to human observers who did not have access to the ex vivo or histopathology data. In comparison to those of the BNN trained with manually annotated ground truth, the proposed method obtained significantly better DSCs with 11% and 16% improvement of calcification and fibrous tissue (Table 3).

### 3.2. Sensitivity to variations in training data

Table 4 shows the DSC between the segmentations yielded by the proposed method when the method was trained with two different training datasets. We observe that the DSC between the segmentations obtained with the two training datasets was greater than 0.8 for all three plaque components with the proposed method. Further, the corresponding DSC values obtained with the U-net-based method were typically lower compared to the proposed method. This provides evidence that the proposed method is relatively insensitive to variations in the training data and more robust than U-Net.

**Table 4:** Sensitivity to variations in training data.

(a) DSC			
	LR/NC with OH	Calcification	Fibrous tissue
Standard U-Net	0.83	0.73	0.76
The proposed method	0.84	0.81	0.85
(b) Precision			
	LR/NC with OH	Calcification	Fibrous tissue
Standard U-Net	0.87	0.77	0.67
The proposed method	0.87	0.85	0.82
(c) Sensitivity			
	LR/NC with OH	Calcification	Fibrous tissue
Standard U-Net	0.80	0.70	0.87
The proposed method	0.87	0.85	0.82

### 3.3. Studying the efficacy of using two networks in the proposed method

The comparison of the proposed method vs. just using a BNN is shown in Table 5. The proposed method significantly outperformed just using a BNN, yielding 15%, 40%, and 19% improvement of DSCs corresponding to LRNC with older hemorrhage, calcification, and fibrous tissue, respectively.

**Table 5:** Comparison of the proposed approach with just using a BNN.

(a) DSC			
	LR/NC with OH	Calcification	Fibrous tissue
One-stage BNN	0.68 (0.65, 0.71)	0.45 (0.41, 0.5)	0.62 (0.58, 0.66)
The proposed method	0.78 (0.75, 0.8)	0.63 (0.6, 0.65)	0.74 (0.73, 0.76)
(b) Precision			
	LR/NC with OH	Calcification	Fibrous tissue
One-stage BNN	0.69 (0.64, 0.73)	0.42 (0.36, 0.48)	0.54 (0.49, 0.58)
The proposed method	0.76 (0.71, 0.8)	0.55 (0.53, 0.58)	0.72 (0.71, 0.74)
(c) Sensitivity			
	LR/NC with OH	Calcification	Fibrous tissue
One-stage BNN	0.68 (0.62, 0.74)	0.48 (0.42, 0.53)	0.72 (0.68, 0.76)
The proposed method	0.8 (0.79, 0.8)	0.71 (0.69, 0.74)	0.76 (0.7, 0.82)

### 3.4. Evaluation with dataset from a different scanner

**Figure 3:**
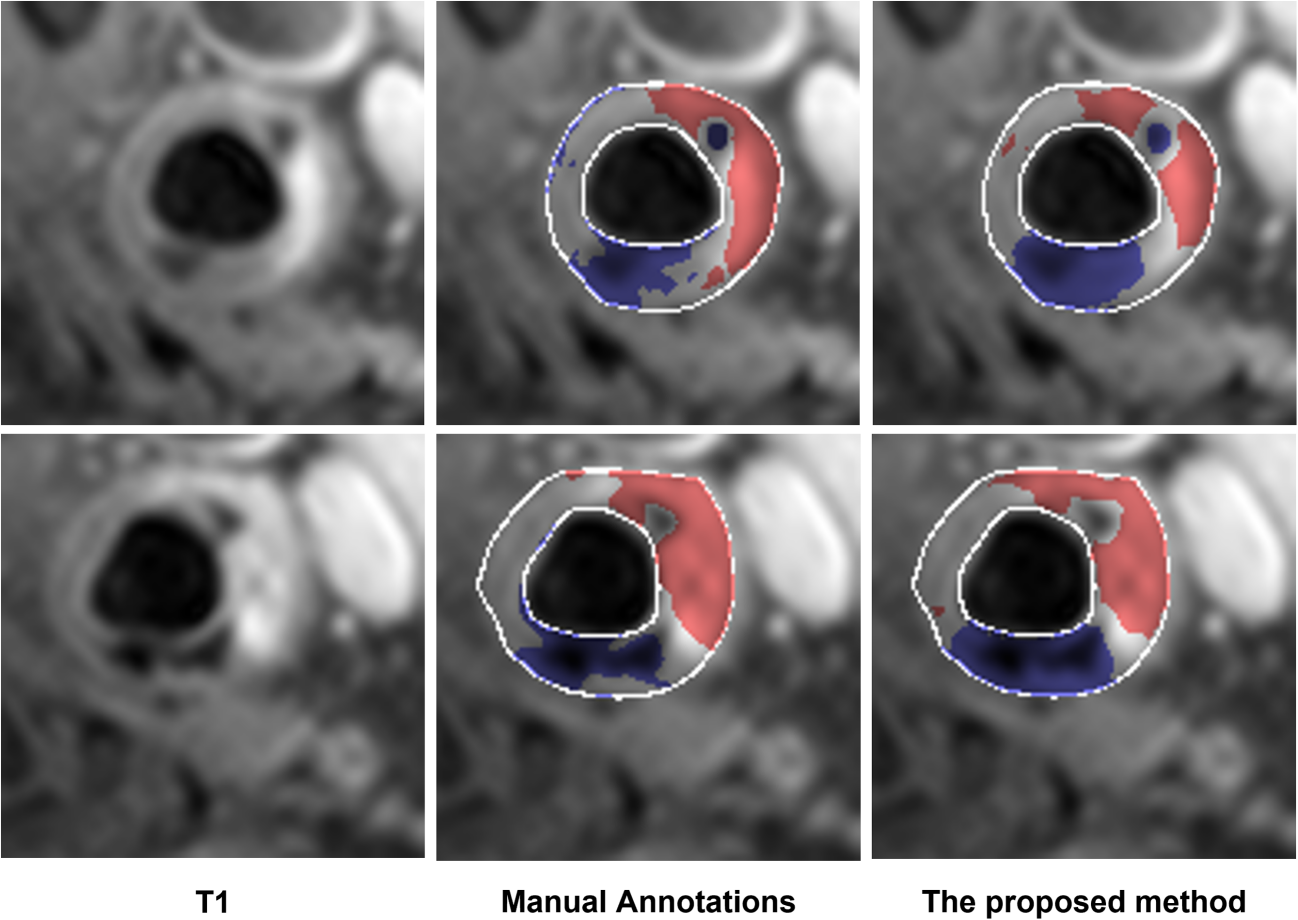
Examples of additional test of the proposed method on the data acquired from the 3 T scanner. The segmented LRNC with older hemorrhage is shown in red, and the calcification in blue, using both the manual annotation and the proposed method. We see that the manual annotations are close to the output obtained with the proposed method.

The performance of the proposed method in the dataset from a 3 T MR scanner (23 patients) is shown in Table 6. The proposed method outperformed Standard U-Net, DeepLab v3, and ResNet-101 methods. In addition, two representative results are shown in Figure 3. In these results, we observe that the proposed method provided segmentation results similar to manual segmentation for both LRNC with older hemorrhage and calcification. We do note that manual labeling classified more tissue as LRNC with older hemorrhage in comparison to our proposed method. However, overall, when assessed upon the same 23-patient MRI data set, our proposed method was more accurate compared to the other three deep-learning-based methods.

**Table 6:** Evaluation with additional test dataset (23 patients scanned on a 3 T MRI).

(a) DSC			
	LR/NC with OH	Calcification	Fibrous tissue
Standard U-Net	0.61	0.54	0.69
DeepLab v3	0.65	0.54	0.67
ResNet-101	0.60	0.53	0.66
The proposed method	0.70	0.6	0.73
(b) Precision			
	LR/NC with OH	Calcification	Fibrous tissue
Standard U-Net	0.79	0.45	0.72
DeepLab v3	0.88	0.42	0.61
ResNet-101	0.76	0.6	0.60
The proposed method	0.78	0.65	0.65
(c) Sensitivity			
	LR/NC with OH	Calcification	Fibrous tissue
Standard U-Net	0.50	0.69	0.67
DeepLab v3	0.52	0.75	0.75
ResNet-101	0.49	0.47	0.74
The proposed method	0.82	0.78	0.88

## 4. Discussion

In this manuscript, based on the hypothesis that a two-stage neural network will account for the class imbalance of vessel wall and background, we implemented a two-stage neural network model with a CNN followed by a BNN to segment carotid atherosclerotic plaque components on multi-weighted MR images. Our major findings are: (1) the ground truth defined with the assistance of histopathology and *ex vivo* MR images improves segmentation performance of deep learning-based method; (2) the performance of the proposed method is superior to other state-of-the-art deep learning methods and manual segmentation by trained readers in segmenting atherosclerotic plaque components on multi-weighted MR images.

Several supervised algorithms for segmenting *in vivo* carotid plaque components in multi-weighted MRI have been developed to facilitate accurate assessment of plaque composition. However, these methods are dependent on manually annotated ground truth through visual comparison between relatively low resolution *in vivo* MRI data and external high resolution histopathological images. This approach may introduce segmentation errors due to the differences in resolution and orientation of MRI and histopathological images. To improve the quality of ground truth, we proposed a strategy where ground truth was generated with the assistance of high-resolution *ex vivo* MR images that were obtained from both non-fixed carotid specimens within 2–3 h after the endarterectomy and histopathology from the then later fixed specimens. As shown in Table 3, this strategy yields superior performance (*p < *0*.*05) in the segmentation of LRNC with older hemorrhage, calcification and fibrous tissue compared to an approach that uses only the manually labeled ground truth for training. The method also provided superior performance (*p < *0*.*05) compared to trained readers who were not provided the high-resolution images. Furthermore, the proposed method outperformed (*p < *0*.*05) all compared deep-learning methods. This improved performance of our proposed method shows the advantages of using ground truth obtained with high-resolution imaging and histology.

We observe in Table 3 that, among all three plaque components, the proposed method yielded the best performance in segmenting LRNC with older hemorrhage. This may be attributed to relatively high sample size of LRNC with older hemorrhage in our data set, providing an abundance of training samples. Moreover, the relatively high contrast of LRNC with older hemorrhage on all 4 contrast-weighted images also contribute to this performance. The fibrous tissue is as common as LRNC with older hemorrhage. However, the lower contrast of fibrosis contributes to a lower segmentation performance compared to LRNC with older hemorrhage. Calcification is hypointense on all four contrast-weighted images, which would make calcification easy to be segmented by our model. In our dataset, calcification was less frequently present compared to LRNC tissue and fibrous tissue. Thus, less accurate performance was observed for segmenting the calcification.

In Table 4, we observe that the method was relatively insensitive to changes in training data samples. This may be attributed to reliable ground truth in the training data sets. More specifically, it is likely that access to high resolution data reduces variability in ground-truth generation, and thus makes the method less sensitive to changes in training data. Another reason may be the probabilistic parameters in BNN, application of ensemble predictors, and resistance of the BNN to overfitting (24). This result also has important practical implications since it implies that the method could be trained at different centers, and still may yield similar performance. Overall, these results provide evidence of the generalizability of the proposed method to additional dataset and to variations in training data.

To assess the generalizability of the proposed method to differences in scanners, we performed additional testing on a separate dataset of 23 patients acquired from a third MRI scanner, which was part of a PET-MRI system. Although this scanner was from the same vendor, the use of a different system and imaging parameters helped evaluate the generalizability of our method. In these 23 patients, the proposed method yielded strong performance (DSC = 0.7) in segmenting LRNC with older hemorrhage (Table 6) demonstrating feasibility of its use in clinical practice.

Recently proposed best practices for evaluation of AI algorithms have recommended that the evaluation of an AI algorithm should yield a descriptive claim that quantifies the performance of the AI algorithm (16). We outline the following claim for the proposed algorithm: “A two-stage neural network-based approach for carotid atherosclerotic plaque segmentation in multi-weighted MRI, that was trained with the assistance of high-resolution imaging and histopathology images, outperformed (*p < *0*.*05) state-of-the-art segmentation methods, yielded DSC of 0.78 (95% CI: 0.75, 0.8) in segmenting LRNC with hemorrhage using an independent test set, and outperformed (*p < *0*.*05) a strategy where the method was trained without the assistance of these high-resolution images.”

Our study had several limitations. The study was performed with data from a single center. To assess for generalizability, evaluation of the method on datasets from different institutions is desirable. Next, our sample size for training the method was limited. To address this issue, we performed data augmentation using flipping and rotation, but other approaches, such as using simulation-based studies (25, 26) may be explored. Next, we note that the proposed method classifies each image pixel as belonging to only one region. However, given the access to high-resolution data, the method could be advanced to compute the volume that a given region occupies in each voxel. A Bayesian partial-volume estimation procedure was recently proposed towards achieving this goal in positron emission tomography (27) and single-photon emission computed tomography (28), and can be advanced for this application. A limitation of the evaluation study with the patient data is that manual segmentation was used as ground truth. However, as mentioned earlier, this segmentation, itself, may be erroneous. Finally, the figures of merit used for evaluation included precision, sensitivity, and DSC, but performance of these metrics may not translate to superior clinical performance (29). In carotid plaque imaging, the clinical goal is to assess vulnerability of the plaque (plaque with a high risk to rupture). Thus, preferably, the method should be evaluated based on this task (30). One challenge in performing this type of evaluation is the lack of ground truth, quantitative values of vulnerability, and lack of correlation to potential patient outcome. To address these issues, no-gold-standard evaluation techniques are being developed that evaluate the performance of segmentation methods on quantitative tasks in the absence of ground truth (31, 32). This research is currently under investigation.

## 5. Conclusion

In conclusion, our proposed deep-learning method trained on ground truth obtained with the assistance of high-resolution *ex vivo* and histopathology data yielded accurate performance of segmentation of carotid plaque components on MR images, outperformed other state-of-the-art segmentation methods, and yielded superior performance compared to trained readers. Additionally, the two-stage neural network model with CNN and BNN architecture was observed to be relatively insensitive to variations in training data and yielded reliable segmentation over other clinical datasets. These promising results motivate further evaluation of the proposed method using larger patient data sets for the accurate assessment of plaque vulnerability.

## Data Availability

The original contributions presented in the study are publicly available. This data can be found here: https://github.com/rockman151/carotid.
