# Supplementary material for: Carotid atherosclerotic plaque segmentation in multi-weighted MRI using a two-stage neural network: advantages of training with high-resolution imaging and histology
Source: Front Cardiovasc Med. 2023 May 24;10:1127653. doi: 10.3389/fcvm.2023.1127653 (PMC10244753; doi:10.3389/fcvm.2023.1127653)
Supplement: Supplementary file 1 [file Table1.docx]

Carotid atherosclerotic plaque segmentation in multi-weighted MRI using a two-stage neural network: Advantages of training with high-resolution imaging and histology

Ran Li, Jie Zheng, Jeffrey E. Saffitz, Pamela K. Woodard, and Abhinav K. Jha

# Two-stage neural network architecture

The convolutional neural network (CNN) and the Bayesian neural network (BNN) had a similar network architecture, comprised of an encoder and a decoder connected with skip connections (Figure 1). The encoder was composed of 5 convolutional blocks with 64, 128, 256, 512 and 1024 output channels, respectively. The first encoder block was comprised of two 3 × 3 convolutional layers with 1 × 1 striding. The other three blocks used two 3 × 3 convolutional layers with 2 × 2 and 1 × 1 striding, respectively, each of them followed by batch normalization and leaky ReLUs. The stride 2 convolutional layer replaced the pooling layer used in standard U-Net models (1). The advantage of the stride 2 convolution layer is that it can learn certain properties that might be ignored in a pooling layer where parameters are fixed during the whole training process. The decoder consisted of 4 deconvolutional blocks with 512, 256, 128 and 64 output channels. These blocks were modified to use 2 × 2 striding transposed convolution followed by a convolutional layer. A concatenation layer that concatenated the up-sample outputs of the last convolutional layers of the four deconvolutional blocks followed the last deconvolutional block of CNN in Stage I. A final 1×1 convolutional layer was used at the end to implement weighted summation regarding the parameters of different filters in the former convolutional layer. To perform estimation of posterior distribution of training data, we replaced the normal convolutional layer with a flip-out convolutional layer in each convolutional block of the BNN in Stage II. The flip-out estimator implemented a stochastic forward pass via sampling from the convolution layer kernel and bias posteriors and carried out Bayesian estimation to the parameters.

Since the contours of the vessel wall were delineated on T1W images only, only T1W images were fed into the CNN in Stage I. The output of the first CNN and the 4-channel images which were comprised of each of the four different weighted MR images in each channel were concatenated as the input to the second BNN structure. The output of Stage II comprised the probabilistic map of the four different components. Training of the two-stage neural network

# Displaying the segmentation output

As the parameters of the BNN are a set of posterior distributions learned on the training dataset, an ensemble estimator with multiple predictors was applied to the output of Stage II. The final segmentation of plaque components was generated by assigning each pixel to the tissue categories with highest probability. To clearly display the segmentation of carotid plaque in this manuscript, all final predictions of tissues were overlaid on T1-weighted images.

# Supplementary Figures


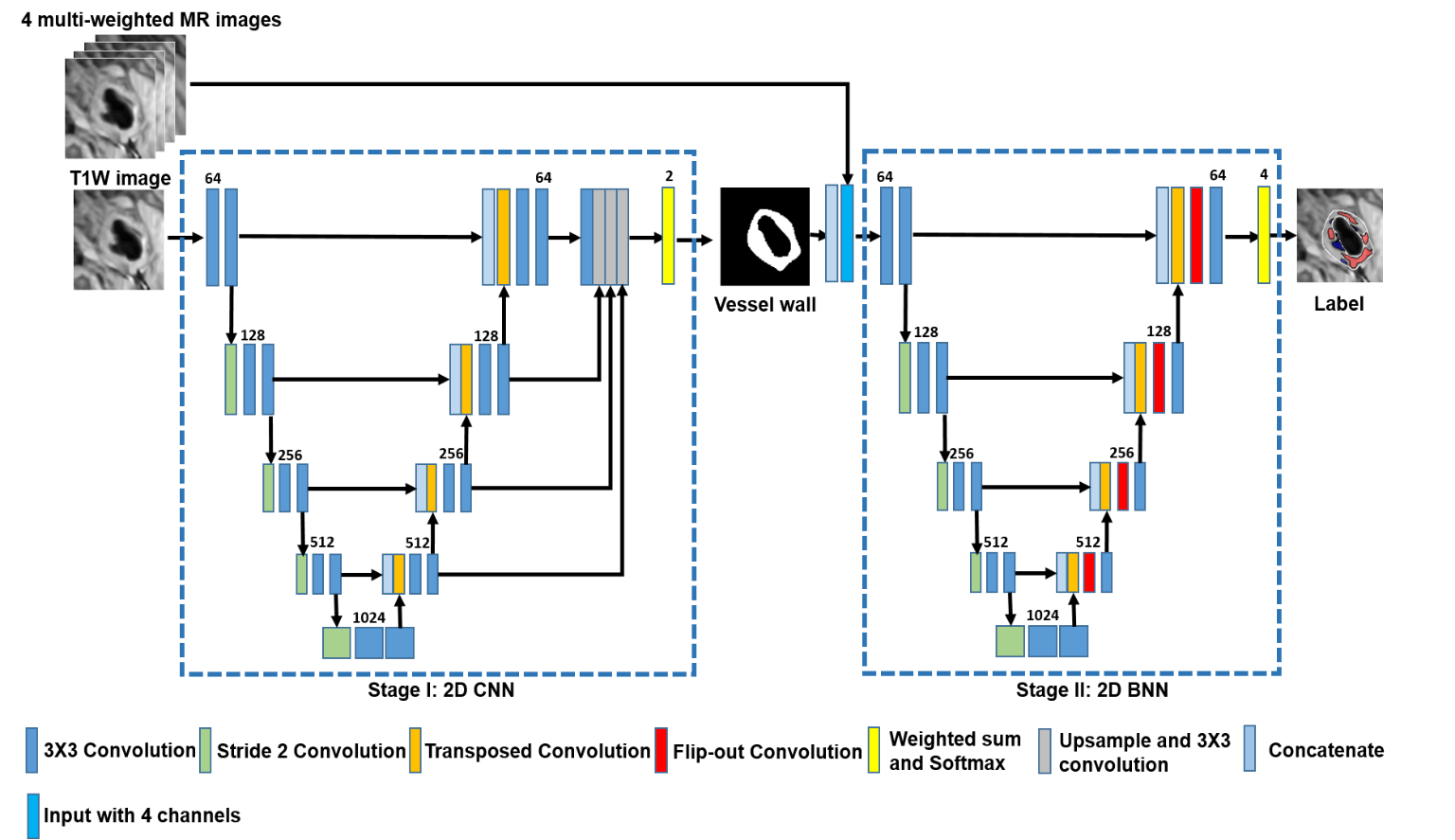


Figure 1: The structure of neural network

References

1. Ronneberger O, Fischer P, Brox T. U-net: Convolutional networks for biomedical image segmentation. Lect Notes Comput Sci (including Subser Lect Notes Artif Intell Lect Notes Bioinformatics). 2015;9351:234–41.
